# Control and maintenance of mammalian cell size

**DOI:** 10.1186/1471-2121-5-35

**Published:** 2004-09-29

**Authors:** Stephen Cooper

**Affiliations:** 1 Department of Microbiology and Immunology, University of Michigan Medical School, Ann Arbor MI 48109-0620, USA

**Keywords:** cell cycle, cell size, exponential growth, linear growth, shift-up, continuum model

## Abstract

**Background:**

Conlon and Raff propose that mammalian cells grow linearly during the division cycle. According to Conlon and Raff, cells growing linearly do not need a size checkpoint to maintain a constant distribution of cell sizes. If there is no cell-size-control system, then exponential growth is not allowed, as exponential growth, according to Conlon and Raff, would require a cell-size-control system.

**Discussion:**

A reexamination of the model and experiments of Conlon and Raff indicates that exponential growth is fully compatible with cell size maintenance, and that mammalian cells have a system to regulate and maintain cell size that is related to the process of S-phase initiation. Mammalian cell size control and its relationship to growth rate–faster growing cells are larger than slower growing cells–is explained by the initiation of S phase occurring at a relatively constant cell size coupled with relatively invariant S- and G2-phase times as interdivision time varies.

**Summary:**

This view of the mammalian cell cycle, the continuum model, explains the mass growth pattern during the division cycle, size maintenance, size determination, and the kinetics of cell-size change following a shift-up from slow to rapid growth.

## Background

Conlon and Raff have described experiments that they claim casts doubt on a basic assumption regarding the way mammalian cell size is maintained during proliferation [1]. The key question studied by Conlon and Raff asks, "How do cells maintain a constant cell size and cell size distribution during extended cell growth?" In a cell culture growing over many generations, the cell size distribution neither varies nor broadens. Cells do not get progressively larger nor do they get progressively smaller. One formulation of this result is that cell mass increase is regulated during the cell cycle so that there is no disparity between the rate of cell mass increase and the rate of cell number increase. Total cell number and total cell mass increase in parallel during unlimited exponential growth. If there were any disparity or disproportion in the rate of mass and cell number increase, cells would get either larger or smaller during extended growth.

In an article accompanying the work by Conlon and Raff [2], a quote by Robert Brooks (Kings College, London) sums up the problem: "If [cell] growth is exponential, then cells must have a size control over division, since otherwise random differences in size at division would increase continuously from generation to generation. This does not happen. Conversely, if growth is not exponential, then such a size control is not necessary." This quote from Brooks may be thought of in this way. Consider two newborn cells of slightly different size. Exponential growth means that cell mass would be made in proportion to the extant cell mass. The larger cell would increase its mass at a more rapid rate than the smaller cell. When the cells divide, the dividing cell produced by the initially larger cell would be even larger compared to the dividing cell produced by the initially smaller cell. Given equipartition of cell mass at division, the new daughter cells would have an even more disparate size difference. Exponential growth in the next cycle would again lead to larger differences in cell size than in the previous cycle. According to this reasoning, the cell size distribution would grow increasingly broader. Since this is not observed, Conlon and Raff propose that either a cell must grow "linearly," or if a cell grows exponentially the cell must have a cell size control system. This reasoning implies that if cells grow linearly, then no cell size control system is required.

The experiments of Conlon and Raff [1] are presented as supporting linear cell growth. Linear cell growth postulates that there is a constant mass increase during each time interval of the cell cycle. Furthermore, comparing their results on mammalian cells to what is referred to as the "yeast" model of cell size control, Conlon and Raff [1] conclude that mammalian cells have a different mechanism for cell size control. As Conlon and Raff summarize their experimental conclusion:

"We show that proliferating rat Schwann cells do not require a cell-size checkpoint to maintain a constant cell size distribution, as, unlike yeasts, large and small Schwann cells grow at the same rate, which depends on the concentration of extracellular growth factors."

A reanalysis of the experiments and reasoning of Conlon and Raff, presented here, leads to a very different view of cell size control and cell size maintenance. It is first shown that there is no problem with either linear or exponential mass increase for size maintenance. Size maintenance does not depend on which pattern of cell mass increase occurs within a cell cycle. The preferred–and experimentally and theoretically supported–pattern of mass increase during the division cycle is exponential growth or exponential mass increase. An exponential growth pattern poses no problem for size maintenance. Constancy of cell size is fully compatible with an exponential pattern of mass increase as well as the hypothetical linear pattern of mass increase. No major difference between the size control systems of yeast, mammalian, or bacterial cells need be postulated to account for the constancy of cell size during the growth of cell cultures. In contrast to the proposed absence of a cell size control system in mammalian cells, it is shown that mammalian cells do have a very simple size control system. The formal elements of this system are similar to that found in the control of the bacterial cell cycle.

## Discussion

### Cell size maintenance with exponential and linear mass increase

**Figure 1:**
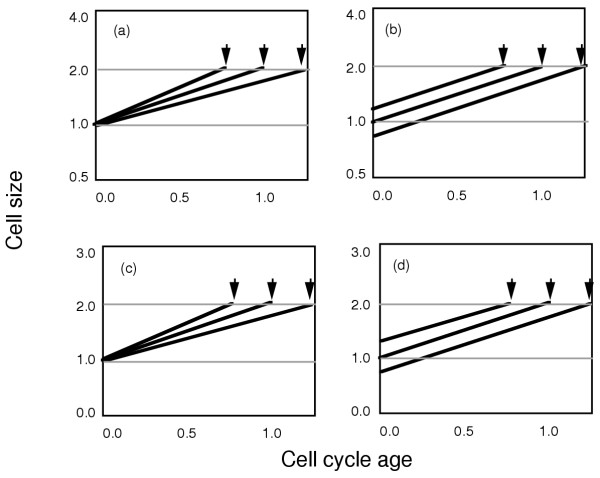
Exponential and linear growth patterns are both compatible with cell size maintenance. In panel (a) newborn cells of identical size increase mass at slightly different rates with an exponential pattern of mass increase. If cells divide at a constant cell size, here size 2.0, size will be maintained even though the rate of mass increase varies. This occurs as the cells divide at different times (division indicated by the downward arrows) as they reach the same size. In panel (b), exponential growth at identical rates from initial cells of different cell sizes also gives size maintenance as cells divide at the same size because there are different interdivision times for each cell; the larger initial cells have a shorter interdivision time and the smaller initial cells have a longer interdivision time. As shown in panels (c) and (d), linear cell increase (note the different ordinate scale compared to panels (a) and (b)) can also lead to cell size maintenance as cells divide at the same size, 2.0.

Can cells grow exponentially during the division cycle and maintain a constant cell size? Consider two possible cases of exponential growth for cells with variable cell sizes. For the first case (Fig. 1a), three cells of the same newborn size have slightly different rates of mass increase. If all three of the cells in Fig. 1a were to have the same interdivision time, the dividing cells would have disparate sizes. But if the interdivision times vary so that cells divide at the same cell size, then cell size is maintained even with exponential growth during the division cycle. A newborn cell that makes mass at a rate slightly faster than average will divide earlier than cells with an average or below average rate of mass increase (Fig. 1a; arrows indicate division times). Conversely, a newborn cell producing mass at a rate slightly slower than average will divide later than cells with an average or above average rate of mass increase (Fig. 1a). Variation in interdivision times allows maintenance of constant average cell size even with exponential mass increase. A second case (Fig. 1b) starts with different sized newborn cells that synthesize mass at the same rate. As in Fig. 1a, the earlier a cell reaches the division size, the earlier the cell will divide and the cell will have a shorter interdivision time. Size constancy is maintained even though mass increases at a constant rate for the three cells with different-sized newborn cells (Fig. 1b). Mixtures of initial size variation and variation of rates of mass synthesis can be analyzed in the same manner; the analysis is strongly supported by a reanalysis of published experimental data on the variation of mammalian cell interdivision times as determined by time-lapse cinematography [3].

Linear cell growth during the division cycle (Figs. 1c and 1d) can also produce size maintenance. Whether cells reach the division size earlier due to a larger initial cell size or due to a more rapid rate of mass increase, the cell size at division can be the same for all cells. Thus size maintenance is also consistent with linear growth.

The patterns shown in Figs. 1a,1b,1c,1d show that there is no impediment to size maintenance as long as interdivision times are not invariant. In all four panels in Fig. 1 the interdivision time varies depending on the time required for a newborn cell to reach a particular cell size.

To be precise, it is not proposed that cells always divide at "exactly" the same size. There is a statistical variation in mass increase and interdivision times that can lead to variations in cell size at division [3]. The important point is that when cells deviate from the mean size there is a return to the mean size through compensating interdivision times during the next cell cycle. Large cells will have a relatively shorter interdivision time, leading to a return to the average cell size.

Further, it is not proposed that a large, newborn cell "controls" its mass increase to have a slower rate of mass increase (compared to smaller cells) thus compensating for the initial larger cell size. Nor do small cells increase their rate of mass synthesis to compensate for their initial mass deficit. Mass increase variation is postulated to have some inherent statistical variation [3] but with all cells, no matter what their extant size, having the same relative rate of mass increase.

There can be variability in cell mass increase with the rate of mass increase being independent of cell size. A large newborn cell could have a faster than average rate of mass increase. In this case, the interdivision time would be even shorter to compensate for both the larger initial newborn cell size and the greater than average rate of mass increase.

**Figure 2:**
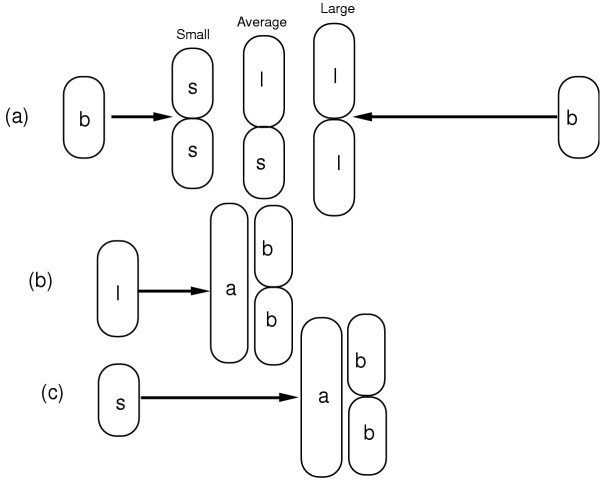
Interdivision time variation allows a return of slightly deviant sizes to a constant cell size. In panel (a) newborn "baby" cells (b) grow for slightly different times, producing either large (l) or small (s) newborn cells from large or small dividing cells. Deviation from equipartition for an average sized dividing cell can also produce large and small cells. The resolution of size differences is illustrated in (b) and (c) where the larger cell (l) has a shorter interdivision time dividing at average (a) cell size and the smaller cell (s) has a longer interdivision time also dividing at average cell size. The dividing average sized cells (a) produce newborn baby cells (b) of the original newborn size.

The size maintenance pattern is illustrated in Fig. 2, where the production of large and small cells can arise either by variation in interdivision times or deviations from equipartition. Newborn or baby cells (b) that have a relatively short interdivision time produce small (s) cells while newborn cells that have a relatively long interdivision time produce large (l) cells. Large and small cells may also be produced by deviation from equipartition at division so that an average-sized dividing cell produces one large (l) and one small (s) cell. The return of small and large cells to the average cell size occurs in the next generation by variation in interdivision times so that small cells (s) have a longer (on average) interdivision time than larger (l) cells (on average).

It may appear that this simple analysis is merely begging the question by not indicating how the cell "knows" to divide at a particular size. This question will be answered below. But first, two issues should be dealt with. An initial discussion will clarify the relationship of cell mass increase to cell size. This will be followed by a discussion of problems with the proposal of linear growth during the division cycle.

### What is meant by the proposal that large cells grow faster than small cells?

What is meant by the Conlon and Raff proposal that, in yeast culture, large cells grow faster than small cells? And conversely, that in mammalian cells, large and small cells grow at the same rate? There are four different meanings that can be given the notion of the rate of mass increase and its relationship to cell size. These different meanings lead to some verbal confusion that requires clarification.

One meaning of the proposal that large cells make mass faster than small cells is that given two cells of disparate sizes, the absolute rate of increase in cell mass is greater in the larger cells. A cell of size 2.0 might add, in some time interval, 0.2 units of cell mass, while a cell of size 1.0, in that same time interval might add only 0.05 units of cell mass. This pattern is a clear and unambiguous difference in the rate of mass increase that is related to cell size.

A second meaning of cell size affecting the rate of mass increase considers that a cell of size 2.0 adds 0.2 unit of mass and a cell of size 1.0 adds 0.1 unit of cell mass over the same time interval. Of course, this case could arguably be said to be a constant rate of mass increase, as the rate of mass increase is proportional to the amount of extant mass. This second proposal is equivalent to mass increasing exponentially. This is because as extant mass changes during the cell cycle the absolute rate of mass increase also changes to reflect the newly added cell mass. After the cell of size 1.0 grows to size 1.1, in the next time interval, rather than 0.1 units of mass being added, there are 0.11 units of mass added to the cell mass. Just as interest is compounded in a bank account, and the funds grow exponentially, so mass in this second example increases exponentially.

A third meaning of the variation in mass increase with cell size is that the rate of mass increase is determined at birth and continues throughout the cell cycle, unaffected by continued cell size increase. A relatively small newborn cell could have a rate of addition of "X" units per time interval, and this rate would remain constant even as the cell increases its cell mass. The larger cell would add more than "X" units each time interval and not change this rate during the cell cycle. This pattern of increase would be called linear synthesis during the cell cycle.

**Figure 3:**
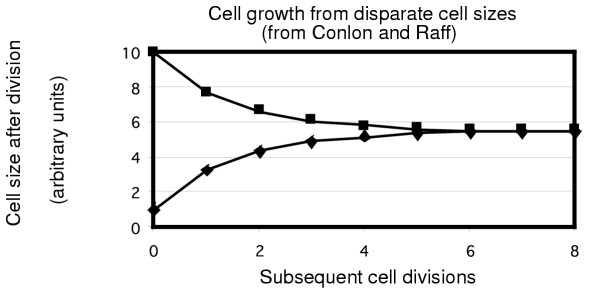
Hypothetical model of Conlon and Raff where constant size increase independent of cell size allows return of deviant cell sizes to a constant cell size over time (This figure is drawn directly from Conlon and Raff (Conlon and Raff, 2003)). The assumption made for this figure is that both large and small cells increase their mass equally over time. Thus, a cell of size 10 and a cell of size 1 increase their mass over one doubling time by 11 units (the sum of the starting masses, 10 and 1). To the large and small cell an increase of 5.5 units of mass is proposed to occur as cells grow. Thus, the large cell grows to size 15.5 and the small cell grows to size 6.5, and at division the daughter cells now have sizes of 7.75 and 3.25 respectively. This continues for a number of generations as the founder cells, originally of disparate sizes, now converge to the same size.

It is interesting to think about these different meaning when considering the theoretical graph drawn by Conlon and Raff [1] to illustrate the return of cells of disparate sizes to the same cell size. As shown in Fig. 3 (redrawn from Fig. 1 of Conlon and Raff [1]), consider two cells, one of size 1.0 and one of size 10.0. During one generation of growth 5.5 units of mass are added by the smaller cell to produce a dividing cell of size 6.5, and 5.5 units of mass are also added to the larger cell to produce a dividing cell of size 15.5. As discussed by Conlon and Raff, upon cell division the daughter cells produced by this pattern of growth would be sizes 3.25 and 7.75. Repeating this each generation (5.5 units added to each cell independent of the extant newborn cell mass) leads, according to Conlon and Raff, to a convergence of cell size as shown in Fig 3.

But no indication of the length of the division cycles is given in Fig 3. If the interdivision times are the same for the large and small cells, which is implicit in, and not excluded by, the analysis in Fig 3 (Conlon and Raff's Fig 1), the relative rate of mass increase for the larger cell is 5.5/10.0 or 0.55 and the relative rate of mass increase for the smaller cell is 5.5/1.0 or 5.5. From this point of view, the ratio of the rates of mass increase is a factor of 10, with the smaller cell making mass from its mass at 10 times the rate (relative to extant mass) compared to the larger cell.

But if the absolute rates of mass increase were the same, then the smaller cell would have a much longer interdivision time than the larger cell. If, over a unit time, 1.0 unit of cell mass were added to the larger newborn cell, and that cell divided at size 11.0, then the interdivision time would be that unit time. The smaller cell, however, would require 10 time units for its interdivision time, because that is the time required to reach size 11.0 as 1.0 unit of material is added to each small cell during each unit of time. The smaller cell grows for a longer time before division. After this first division the new daughter cells produced by each of the initial cells would be the same size. By allowing interdivision time variation, cell size uniformity is restored in one generation.

A similar analysis can be made for exponential mass increase (i.e., mass added proportional to extant mass). If the cell of size 10.0 added 1.0 unit of mass in a unit of time, then the small cell would add 0.1 unit of mass in that same time interval. In this case, there would be even more time required for the small cell to reach the division size of 11.0. In any case, exponential growth coupled with interdivision time variation can allow size maintenance because both the large and the small newborn cells will divide at the same size, as described in Fig. 1.

Of course, the example given by Conlon and Raff (Fig 3) as discussed here is unrealistic. Cell sizes do not vary over a factor of 10 in exponential culture. But this re-analysis of Fig. 3 illustrates the power of considering different interdivision times as a factor in maintaining constant cell size.

Robert Brooks (personal communication) notes that in some of his experiments cell size is observed as very variable. He states that in experiments with Shields they found that size varied over a range of at least 6-fold. In response it can be pointed out that recent careful measurements of the size variation during the division cycle of cells grown under ideal conditions indicates that size variation is not broad [4-7]. Helmstetter (personal communication) points out that when cells are not grown under optimal conditions there are always some cells of odd or abnormal size. But these cells are cells that are dying or in some way impaired. These abnormally sized cells should not be considered as typical of the cells in a well-maintained, exponentially-growing cell culture.

The fourth part of our verbal analysis of mass increase as a function of cell size relates to bacterial cells. As will be seen below, one of the most important results in bacterial physiology is that as growth rate speeds up, cells get larger. As the growth rate of a cell is continuously varied by increasing the richness of the medium, there is a continuous variation in bacterial cell size with the faster growing cells being larger than slower growing cells [8]. Bacterial cells with an interdivision time of 20 minutes are larger than cells with an interdivision time of 60 minutes. Although it could be said that larger cells make mass faster leading to the shorter interdivision time for larger cells, it is equally possible, and in fact preferred, to reformulate or verbalize this result by saying that faster growth produces larger cells. For a given medium the rate of mass increase is determined for the bacterial cells, and the cell size *results *from the growth rate. This idea, the fourth way of looking at the relationship of cell size and mass increase, will be illustrated below in the analysis of bacterial patterns of DNA replication and cell size maintenance.

As we shall see, in bacterial cells a constant period for DNA replication and a constant time between termination of replication and cell division explains the variation in bacterial cell size as a function of growth rate. This same explanation also applies to mammalian cells: the rate of growth determined by external conditions determines cell size. Rather than taking the results of Conlon and Raff and concluding that larger cells when placed in medium with more serum now grow faster, it is better, as with bacteria, to say that when cells are placed in a condition that provides faster growth (i.e., a shorter interdivision time), the cells grow larger. While this may appear, at first sight, to be a trivial and semantic difference, this distinction actually lies at the heart of the problem and is the key to the solution of size determination and size maintenance. Rather than thinking that cell size produces cells with a particular growth rate (e.g., large cells grow fast), it is preferable to think that a particular growth rate produces cells of a particular size (e.g, fast growing cells are made larger than slower growing cells).

### What is wrong with linear growth?

There are problems inherent and unavoidable in any proposal of linear cell growth during the division cycle. Linear growth means that during the division cycle, as a cell proceeds from size 1.0 to size 2.0, cell mass is added at a constant amount per unit time. If a cell grows linearly, over tenths of a cell-cycle time, a cell increases its size from size 1.0, to 1.1, to 1.2, and so forth.

The main problem with linear growth (i.e., constant amounts of cell mass are added at constant time intervals) is that as the cell gets larger, the cytoplasm becomes inefficient. Inefficiency is defined here as producing less mass per extant mass compared to more efficient use of the extant mass. Efficient mass increase would exist when extant cell mass makes new mass as fast as possible. As a cell grows, more cytoplasm is present. With linear growth the extra cytoplasm does not increase the absolute rate of cell mass synthesis. In essence, the new cytoplasm does not work to make new mass. There is a decrease in the relative rate of mass increase (i.e., mass synthesis per extant mass) which means that the ribosomes, after some growth, are not working as efficiently as before there was growth.

One mechanistic model explaining the postulated absence of a change in the absolute rate of mass increase to produce linear growth is to propose that the new mass does not enter into active participation in mass synthesis until a cell division; new mass will not be "activated" to enter into mass synthesis until the next division. From this viewpoint, there is a constant rate of mass increase based on the original mass. As a cell approaches division, the efficiency of mass making new mass tends toward half that of the efficiency of the initial, newborn cell mass. An alternative mechanistic proposal to explain linear growth is that during a cell cycle the amount of material able to be taken up by a cell is constant, and only upon cell division is there an "activation" of the new cell surface so that there is an increase in the ability of the cell to take in material.

Even more important and troublesome is the result that if a cell grows linearly, at the instant of cell division there must be a sudden saltation or jump in the synthetic activity of the cytoplasm. Toward the end of the cell cycle, 1.9 units of cell mass make 0.1 unit of cell mass to achieve a cell mass of 2.0. Given linear growth, at the instant of division the 2.0 units of cell mass, now apportioned into two daughter cells, must now make, during the next time interval, 0.2 units of cell mass or twice as much as in the previous time interval. When the cell of size 2.0 divides, linear growth implies that the two new daughter cells now immediately activate the "quiescent" cytoplasmic material (or activate the previously inert cell surface uptake capabilities). Irrespective of mechanism, considering the two daughter cells together, linear growth during the division cycle inevitably implies that at division there is a sudden doubling in the rate of mass increase.

There is no known biochemical mechanism for these proposals to produce linear cell growth, or the sudden jump in the rate of mass increase. As currently understood, the new cytoplasm joins right in to make new mass. And there is no mechanism known to allow new cell surface to remain inert until a cell division. While the absence of any identification of these mechanisms does not mean that these mechanisms do not exist, there is no need to propose the existence of these mechanisms if cells grow exponentially.

The experimental evidence favors exponential mass increase during the cell cycle. In bacteria the evidence for exponential growth is extremely strong [9,10]. Analysis of data on eukaryotic cell size increase also supports exponential growth during the division cycle [11].

What of the experiments presented by Conlon and Raff [1] that cell mass increases linearly? Conlon and Raff studied cells cultured in 1% fetal calf serum, forskolin, and aphidicolin. Aphidicolin is an inhibitor of DNA synthesis. While mass increased, there was no concomitant increase in DNA. The cells were incubated for 216 hours (9 days). The cell volume was measured using a Coulter Counter, although in one experiment total protein content was measured.

Conlon and Raff realized that it is extremely difficult to distinguish linear from exponential growth over one doubling time. Therefore they measured mass increase over a longer period of time (approximately 3 or more normal interdivision times). The problem with this experiment is that the inhibited cells do not allow an exponential increase in cell number as DNA synthesis is inhibited. Therefore the experiment is subject to the critique that aphidicolin inhibition produced the observed results. The results may not, and very likely do not, reflect the situation in normal, uninhibited, and unperturbed cells. For example, there could have been exponential growth during the first "virtual cell cycle". Then the limitations of DNA content would lead to the observed linearity of growth as measured over the extended period of analysis. But this linearity should not be taken as an indication that during the normal cell cycle the cell mass increases linearly.

**Figure 4:**
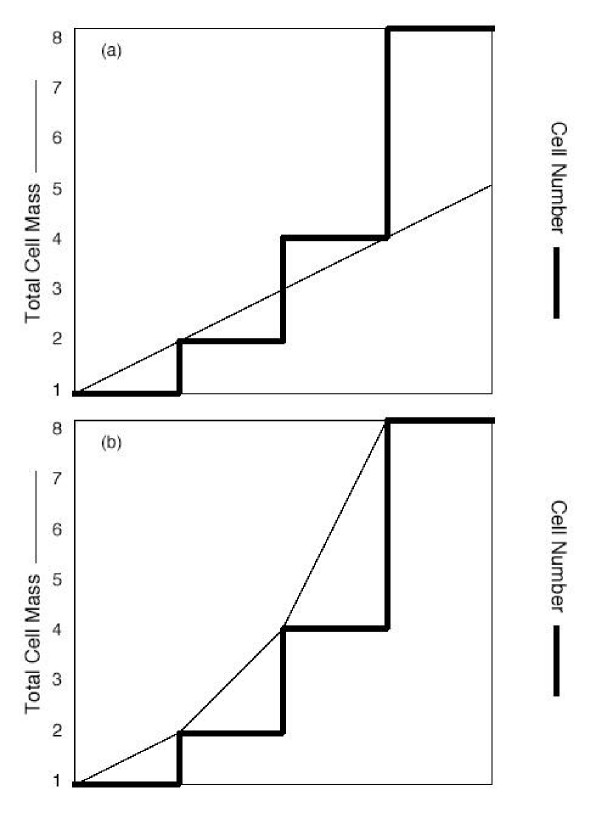
Approach of cell mass to exponential even if cells had linear synthesis within cell cycle. Panel (a) illustrates cells dividing to produce two, four or eight times the original number of cells (thick line is cell number). The mass (thin line) increases linearly. It is clear that the cell size will not be maintained. In panel (b), even with linear mass growth within the cell cycle (thin line), as cells divide the rate of mass synthesis doubles and then quadruples as cell numbers increase. It is not proposed that mass increases linearly, but merely that even linear synthesis should exhibit, in an uninhibited situation, exponential mass growth.

Even if cells grow linearly during the division cycle, if the rate of mass increase is measured over a number of cell cycles with uninhibited cells, then *a priori *there should be evidence of an approach to exponential mass increase. If the rate of mass increase during the first cycle is 1.0, during the second cycle it should be 2.0, during the third cycle 4.0, and so on. Thus, even on its own terms, with linear mass increase during the division cycle, the experiments of Conlon and Raff [1] on the pattern of mass increase are flawed by the presence of an inhibitor of DNA synthesis. An analysis of this idea is presented schematically in Fig. 4.

Raff (personal communication) disputes this interpretation of the aphidicolin experiments, proposing that "while the aphidicolin-arrest strategy is certainly artificial, it is not unrealistic...as many cells, including Schwann cells, grow a great deal after they have stopped dividing. Moreover...hepatocytes grow linearly, independent of their size, if a mouse is re-fed after it has been starved for a couple of days." As noted in Fig. 4, without inhibition, growing cells that grow and divide must, *a priori*, approach an exponential pattern (i.e., rate of 1, to 2, to 4, to 8 as cells multiply), and therefore the only meaningful discussion of the linear vs. exponential growth pattern relates to growth within the cell cycle. Regarding application of liver growth following starvation and refeeding, this complex situation seems particularly inapplicable to discussions of cell growth in cell culture as there are so many complicating factors. A detailed analysis of the proper systems for cell-cycle analysis has been presented [4].

The experiments of Conlon and Raff also show some internal inconsistencies that weaken the actual data. A comparison of cell volume increase and protein per cell increase in the same cells over a 96 hour period (Fig.3 of Conlon and Raff) shows that the volume increase was 4.75-fold (~2,000 μm^3^/cell increasing to ~9,500 μm^3^/cell) but the protein increase was only 2.93-fold (~0.16 ng/cell increasing to ~0.47 ng/cell). Until these differences are resolved, it is difficult to accept these experiments as supporting linear cell growth–or any other pattern of cell growth–during the normal division cycle. The discrepancies pointed out here suggest that the quantitative measures of cell size by Coulter Counter may not be able to distinguish different growth patterns.

Another problem arises in Conlon and Raff's [1] analysis of the pulse-chase experiments where cells starved for different times are pulsed and chased to measure protein turnover. They concluded: "...the rate of decrease in radiolabeled protein increased as the cells increased in size." That is, there was a greater release of labeled amino acids from cells that were inhibited with aphidicolin for longer periods of time and which were therefore larger [1]. But the release data were plotted on rectangular coordinates. This led to the observation that the slope between the 0 hour and 2 hour points in their Fig.4 is steeper for the cells arrested for 72 hours compared to the cells arrested for 48 hours. The 72 hour cells were larger than the 48 hour cells. But considering the actual values, and reading the results from the published graph, the counts for the 72 hour arrested cells went from ~179 to ~121 in two hours, or a ratio of 0.67 for the two hour chase. The 48 hour arrested cells went from ~138 to ~94 for a ratio of 0.68 for the two hour chase. Thus, in contrast to the conclusion of Conlon and Raff [1] there is no apparent difference in the turnover of proteins as a function of cell size.

Robert Brooks (personal communication) has argued against this analysis, noting that the cells starved for 24 hours appeared to show "no turnover" as the line for this graph (Conlon and Raff's Fig.4) was flat. But in the text in the legend to their Fig.4(b) Conlon and Raff state, "The shallowness of the curve for the 24-hour-arrested cells is likely to be the result of the lower than expected value at 0 hours." This explanation comes from the initial counts in Fig.4(a) where it can be seen that there is some apparent error in the zero time value for the 24 hour starved cells in their Fig.4(b).

**Figure 5:**
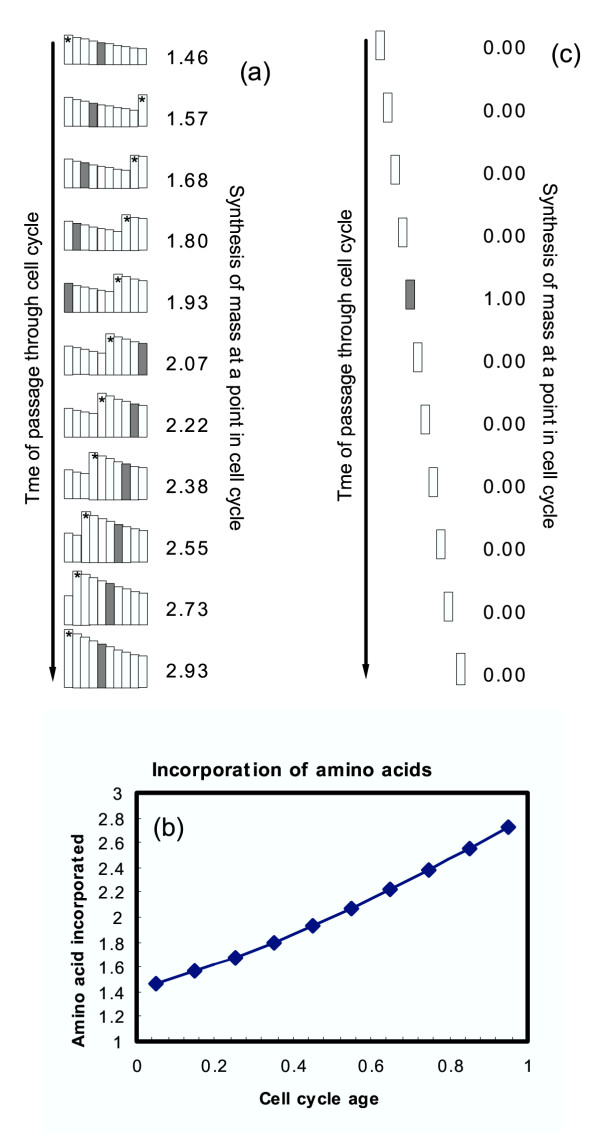
Unsynchronized cells cannot be used to determine cell-cycle pattern of synthesis. Panel (a) shows a series of age distributions starting with the initial age distribution reflecting the pattern Age Distribution = 2^1-X^, where X is the cell age going from 0.0 to 1.0. In this *Gedanken *analysis, it is assumed that cells of age 0.5 (i.e., cells in mid-cycle) are the only cells incorporating amino acid (cross-hatched bars). The asterisk (*) on a bar in each pattern indicates the newborn cells. One reads the cell ages by going from the asterisked bar to the right and then back to the left to finish off the age distribution. The number to the right of each pattern is the relative number of cells incorporating amino acid. Thus, in the uppermost pattern in Panel (a) the relative number is 1.46. After one-tenth of a generation we see that the oldest cells in the first pattern have divided to give double the number of cells and these cells are now the youngest cells in the culture. All of the other cells move up one-tenth of an age so that the cells that were age 0.4 are now age 0.5 (cross-hatched bar) and the rate of synthesis increases to 1.57. This is because there are more cells in the original culture of age 0.4 than there were of age 0.5. Continuing down the patterns in Panel (a) we see that as cells move to age 0.5 there is a continuous, and exponential, increase in the radioactivity. The cells above age 0.5 (in the original topmost diagram) divide and produce two cells each tenth of a cell cycle, so that over one total cell cycle there is an exponential increase in the rate of amino acid incorporation (a measure of cytoplasm increase). The total pattern of incorporation is plotted in panel (b) where the exponential incorporation during one cell cycle is indicated. Panels (a) and (b) thus show that even with a non-exponential pattern of incorporation, if a total culture is studied, the measured incorporation pattern will be exponential. If, however, cells are truly synchronized, as illustrated in Panel (c), a peaked incorporation pattern is observed, accurately reflecting the mid-cycle incorporation of amino acids into the cells at a particular cell-cycle age. Starting with newborn cells at age 0.0 and moving through the cell cycle at one-tenth of an age each pattern in (panel c) the incorporation (noted by the numbers to the right of the diagrams (panel c) shows a peaked pattern.

But an even more egregious error in analysis precedes even these technical problems. The cells studied by Conlon and Raff were not synchronized. The cells were not aligned and were in all phases of the cell cycle. Theoretically, it is impossible to determine the pattern of mass synthesis during the cell cycle on cells that are not synchronized. (For complete details see [12]). This is because of the age distribution of cells in a growing culture. The age distribution for growing cells in culture is given by 2^1-X^, where X is the cell age during the cell cycle; X varies between 0.0 and 1.0 (newborn cells are age 0.0 and dividing cells are age 1.0). At age 0.0 the relative number of newborn cells is 2.0 (2^1-0 ^= 2^1 ^= 2) while the relative cell number of dividing cells is 1.0 at age 1.0 (2^1-1 ^= 2^0 ^= 1). This distribution of cell ages means that any incorporation measurement on asynchronous cells must, and will, yield an exponential pattern of uptake. This is illustrated in Fig. 5 for an idealized case where we imagine cells making all of their cytoplasm only at age 0.5. Because of the age distribution an exponential pattern of incorporation is observed when the entire culture is analyzed (Fig. 5a,5b). The details of the analysis are presented in the legend to Fig. 5. If the cells had been synchronized then one would have measured a peaked pattern as illustrated in Fig. 5c.

Robert Brooks (personal communication) argues that this critique is incorrect because "they [1] started with quiescent (G0/G1) cells." Quiescent cells with a G1-phase amount of DNA are not synchronized [13-15]. The reader is referred to these papers for a detailed analysis. Despite the widespread belief and acceptance that cells can be synchronized by growth arrest (i.e., by whole-culture synchronization methods), this idea is incorrect. Cells can only be synchronized by selective methods [15].

**Figure 6:**
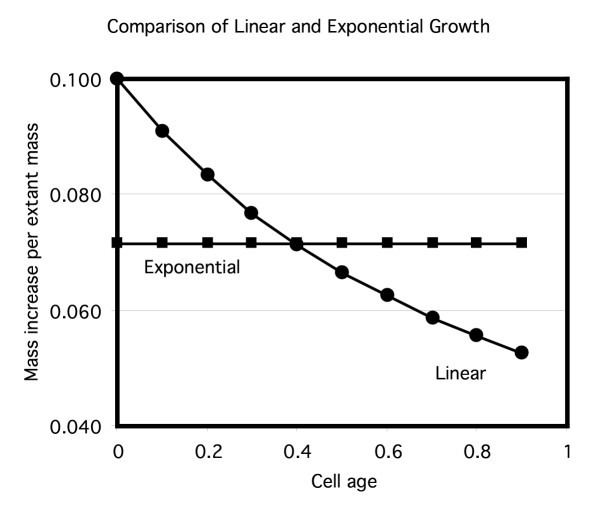
Comparison of the ratio of pulse label to total label for exponential and linear patterns of mass increase as described in Table 1.

How can one determine whether mass increases exponentially or linearly during a normal, unperturbed, division cycle? To illustrate one approach to determining the pattern of mass increase during the division cycle, consider the following experiment. Grow cells for many generations in a radioactive amino acid (e.g., C-14 labeled amino acid) so that cell protein is totally labeled. Then add a pulse of a counter-labeled amino acid (e.g., H-3 labeled). As shown in Table 1, if cells grow linearly, the ratio of tritium (H-3) to C-14 should decrease as the cells become larger. With exponential growth the ratio of tritium to C-14 should be constant over the cell cycle. If one now one took such double-labeled cells, fixed them, and spread the cells out on a gradient such that the larger cells were preferentially at the bottom and the smaller cells at the top, if cells grew linearly there would be a decrease in the H-3/C-14 ratio as the larger and larger cells were assayed. If cells grew exponentially there would be a constant radioactivity ratio over the entire set of cell size fractions. The idealized results from Table 1 are illustrated in Fig. 6.

**Table 1:** Analysis of linear and exponential growth by comparing long-term and short-term isotope incorporation.

LINEAR GROWTH				EXPONENTIAL GROWTH		
Cell size	Size increase	Inc/size	**Cell age**	Cell size	Size increase	Inc/size
1	0.1	0.100	**0**	1.00	0.07	0.072
1.1	0.1	0.091	**0.1**	1.07	0.08	0.072
1.2	0.1	0.083	**0.2**	1.15	0.08	0.072
1.3	0.1	0.077	**0.3**	1.23	0.09	0.072
1.4	0.1	0.071	**0.4**	1.32	0.09	0.072
1.5	0.1	0.067	**0.5**	1.41	0.10	0.072
1.6	0.1	0.063	**0.6**	1.52	0.11	0.072
1.7	0.1	0.059	**0.7**	1.62	0.12	0.072
1.8	0.1	0.056	**0.8**	1.74	0.12	0.072
1.9	0.1	0.053	**0.9**	1.87	0.13	0.072
2			**1**	2.00		

The center, bold-faced, column lists the cell ages from 0 to 1.0. At the left the linear increase of mass is related to the absolute increase in mass per interval (0.1 each interval for linear increase in mass during division cycle), and the ratio of incorporation per extant cell mass is given in the third column (0.1 to 0.053). Similar results for exponential growth except the mass increase per interval goes from 0.07 at the start of the division cycle to 0.13 at the end. The ratio of incorporation per extant mass in the right-most column is thus constant.

To summarize this critique of the aphidicolin-inhibition results, the experiments of Conlon and Raff do not measure the mass increase during the cell cycle. The experiments using inhibition of DNA replication merely measure the pattern of mass increase in a perturbed experimental situation on cells that are not synchronized. This experiment is not supportive of any particular pattern of mass increase during the normal division cycle. More important, as shown in Fig. 5, without synchronization of cells, it is impossible to determine the pattern of mass increase during the division cycle.

### The bacterial cell cycle: Rules, patterns, and regulation

This analysis presented here explicitly deals with animal or eukaryotic cells. However, it is relevant to bring to bear on this problem the experience and results obtained regarding cell-size determination in bacteria. In 1968 the rules for the replication of DNA in a simple bacterium (*Escherichia coli*) as well as the relationship of cell size to control of DNA replication were worked out [16-20]. The pattern of DNA replication and cell size are determined by three rules:

1. A round of DNA replication is invariant (40 minutes) over a wide range of growth rates [16-19,21].

2. The time between termination of replication and cell division is invariant (20 minutes) over a wide range of growth rates [16-19,21,22].

3. At the time of initiation of replication, the cell mass per origin is a constant [16,20,23].

**Figure 7:**
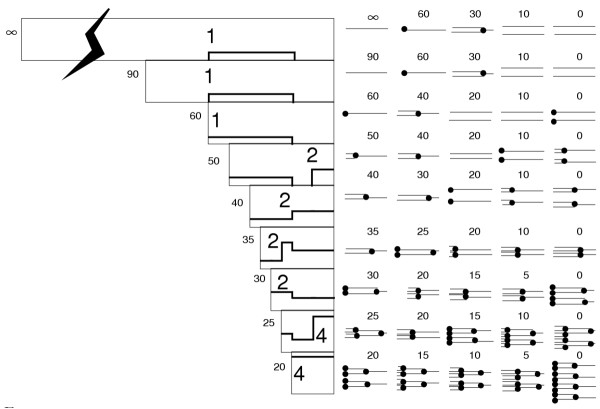
Diagram of patterns of DNA replication during the division cycle in bacteria. The different patterns go from an infinite interdivision time (i.e., essentially no or extremely slow growth) to cells with 90, 60, 50, 40, 35, 30, 25, and 20 minute interdivision times. In all cases, the rate of replication fork movement is 40 minutes for a round of replication or one-quarter of the genome every 10 minutes. All rounds of replication end 20 minutes before the end of the cell cycle. This is most clearly seen in the 60-minute cells where a newborn cell has one genome, which replicates for 40 minutes ending replication 20 minutes before cell division. The same rules are drawn here for a 90-minute and a very slow growing cell (infinite interdivision time). The large numbers in each pattern at the left indicate the number of origins to be initiated at each time of initiation of replication. Thus, in the 60-minute cells there is one origin in the newborn cell. Consider that the cell mass is given a unit value for each origin to be initiated. Thus, the newborn cell in the 60-minute case is given a size of 1.0 unit of mass. This means that the dividing cell in the 60-minute cells is size 2.0. Mass increases, in the 60-minute case, from 1 to 2. In the 90-minute cells the cell of size 1 is one third of the way through the cell cycle. Since mass increases continuously during the division cycle it is clear that the newborn cell in the 90-minute culture is less than 1.0 in size. Let us say it was something like size 0.7. In this case the newborn cell in the 90-minute cells would be size 0.7 and the dividing cell would be size 1.4. It is clear that the 90-minute cells are, on average, smaller than the 60 minute cells. Similarly, if we consider the very slow cells, the cell of size 1.0 is very near the end of the cell cycle, and the newborn cell is slightly above size 0.5. Since the very slow growing cells (top panel) go from sizes 0.5 to 1.0 and the 60 minutes cells go from size 1.0 to 2.0, the 60 minute cells are twice as large as the very slow growing cells. The 30-minute cells have two origins in the newborn cell and thus the newborn cells can be considered size 2.0 with the dividing cells 4.0. The 20-minute cells have a newborn cell of size 4.0 (four origins in the newborn cell) and a dividing size of 8.0. As one goes from extremely long interdivision time, to 60, to 30 to 20, the relative sizes go from 0.5, to 1, to 2 to 4, with the growth rates expressed as doublings per hour, or 0 (infinite interdivision time), 1 (60 minute interdivision time), 2 (30 minute interdivision time), and 3 (20 minute interdivision time). Cells that initiate DNA replication in the middle of the cycle may be considered as follows. The 40-minute cell has two origins in the middle of the cell cycle so the mid-aged cell is size 2.0. The newborn cell might be some size like 1.5 and the dividing cell something like 3.0. Thus, the 40-minute cell has an average size intermediate between the 60 and the 30-minute cell. Similarly, the 25-minute cell also initiates mid-cycle, but there are 4 origins at the time of initiation. Thus, the mid-aged cell in this case is size 4.0 and the newborn cell may be considered something like size 3.0. The cell sizes go from 3.0 to 6.0, and these cells are larger than the 30-minute cells and smaller than the 20 minute cells.

**Figure 8:**
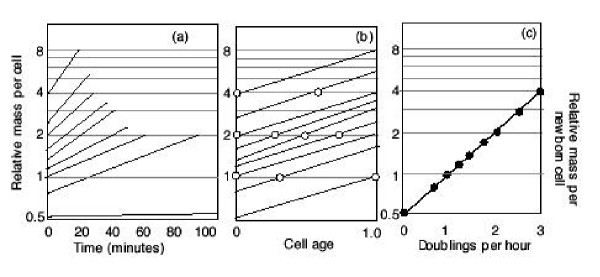
Size determination in bacteria. In panel (a) the rates of growth of cells from infinitely slow (very long interdivision time) minutes to 20 minutes (as illustrated in Fig. 6) are plotted with the relative sizes shown. Thus, the 60 minute cell goes from size 1.0 to 2.0 over 60 minutes. The 30-minute cell (third angled line from top) goes from size 2 to 4 over 30 minutes. And the 20-minute cell (top angled line) goes from size 4 to 8 over 20 minutes. Other rates of growth for 25, 35, 40, 50, 90 and "infinite" interdivision times are also shown. In panel (b) the same results are plotted over relative cell ages from age 0 (newborn) to 1.0 (dividing cell). The open circles indicate when initiation occurs, and corresponds to the numbers in the individual panels. Thus, in Fig. 6 the cells with a 60, 30, and 20 minute interdivision time initiation DNA replication in the newborn cell (age 0.0) at sizes 1, 2 and 4. Besides the cell age at initiation, the open circle also indicates the relative size of the cell at initiation (see numbers in Fig. 7). The cell sizes at age 0.0 for all cells is a measure of the average cell size in the culture. (Given an identical pattern of cell growth during the division cycle the relative cell size of the cells in a culture is precisely proportional to the newborn cell size). These size values are then plotted against the rate of cell growth (the inverse of the interdivision time or doublings per hour) as shown in panel (c). The log of the cell sizes are a straight line when plotted as a function of the rate of cell growth (the inverse of the interdivision time).

These rules are illustrated in Figs. 7 and 8. These three rules predict (Fig. 8c), that cell size should be a logarithmic function of growth rate. Cell size plotted on semi-logarithmic coordinates against the reciprocal of the interdivision time (i.e., the growth rate) gives a straight line. Faster growing cells are larger than slower growing cells. Ten years earlier, in 1958, before the rules predicting the size-growth rate relationship were determined, this experimental result [8] was clearly obtained in what has been called "the Fundamental Experiment of Bacterial Physiology" (Cooper, 1991)[12]. An analysis of the history, origins, and meaning of this experiment has been published (Cooper, 1993)[40].

The important consequence of Figs. 7 and 8 is that we understand how cell size is controlled in bacteria. Cells initiate DNA replication at a certain cell size. This cell size (sometimes referred to as the "initiation mass") is a constant size within experimental limits (Cooper, 1997)[23]. The cell size at initiation is constant per origin present in the cell. A cell with two origins being initiated is twice as large as a cell with only one origin. The number of origins present at initiation and the cell age during the division cycle at which initiation occurs determines the average cell size of a cells growing in culture.

### Analysis of size maintenance in animal cells

**Figure 9:**
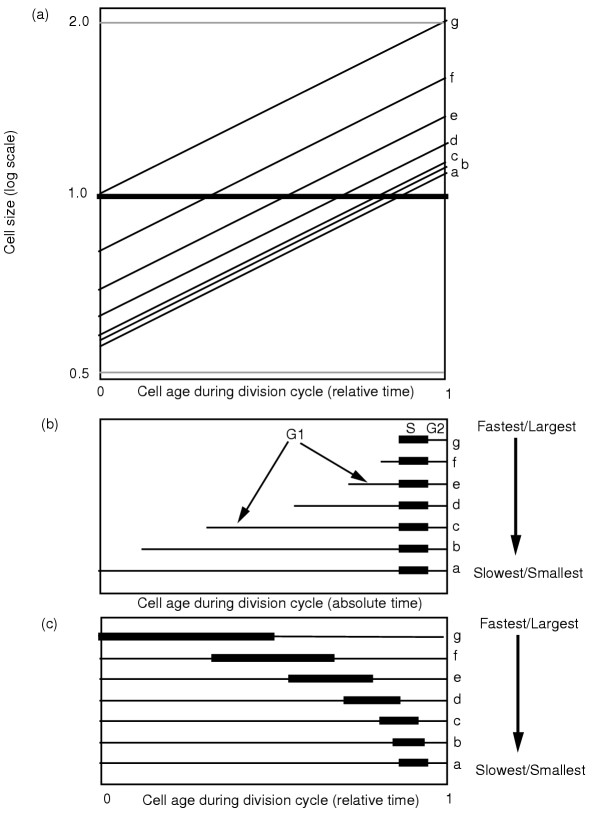
Mammalian cell size variation as growth rate varies. Panel (a) shows a given mammalian cell growing at different rates and with different sizes. The lines are parallel because the interdivision times are normalized to a relative cell age as cells are born at age 0.0 and divide at age 1.0. All lines are exponentially increasing cell sizes from smallest to largest. Where the lines cross the thick horizontal line indicates a cell of size 1.0. Since the fastest cell (cell g) has a size 1.0 at the start of the cell cycle these cells must go from a newborn sizes of 1.0 to a size at division of 2.0. The slowest cell (cell a) has size 1.0 toward the end of the cell cycle, so the newborn cell is slightly larger than size 0.5 at age 0.0. The size ranges of these cells goes over a factor of 2. In panel (b) the size patterns are re-interpreted in terms of initiation at a particular time during the cell cycle. In this figure the thick, short line on each pattern is the S phase, the thinner line to the right is the G2 phase and the thinner line to the left is the G1 phase. Given that S and G2 are relatively constant in length then the slower cells (e.g., cell "a") have a longer G1 phase than the faster growing cells (e.g., cell "g", which has no measurable G1 phase). This is because the interdivision time is the sum of S+G2+G1. If S and G2 are relatively constant as the interdivision time decreases (i.e., as cells grow at faster growth rates), the G1 phase gets smaller. When the interdivision time equals the sum of S and G2 as in cell "g", there is no G1 phase. Such a situation has been analyzed previously (Cooper, 1979). It is clear from panel (b) that as cells grow faster, the time during the division cycle at which initiation of S phase starts is earlier and earlier. This is illustrated even more directly in panel (c) where the phases are normalized to a unit length. The slowest cell (cell "a") has the shortest fraction of cells with an S or G2 phase and the fastest growing cell (cell "g") has the entire division cycle occupied by S and G2 phases. The topmost line in panel (c) is the fastest cell and it starts S phase early in the cell cycle. Thus we see that the faster a cell grows the earlier in the cell cycle the cell achieves a size of 1.0. This accounts for the result that the slower cell has a smaller cell size than the faster growing cell.

The ideas of the bacterial cycle can be directly applied to animal cells. Cells of different growth rates are shown in Fig. 9a. The different lines, a-g, identify cells of different sizes because they pass through size 1.0 at different cell ages during a cell cycle span. Cell "g" is a faster growing cell than cell "a" with the others of intermediate growth rates. The earlier a cell reaches size 1.0, the larger the cells will be. Thus, in Fig. 9a, the cell "g" is larger than the cell "a" because the cell "g" reaches size 1.0 earlier than the cell "a". As drawn in Fig. 9a, the newborn "g" cell is size 1.0. The mother or dividing cell is size 2.0. We can imagine that the mean size of cells growing at this rate is approximately 1.5. In contrast, the "a" cell varies between newborn size of approximately 0.6 and dividing size of 1.2. The average size of the "a" cells is smaller than the "g" cell, approximately size 0.9. (The precise calculation of the average cell size requires consideration of the age distribution and the actual pattern of mass increase during the division cycle; for purposes of this analysis, these complications are omitted.) Other cells (b-f) may be similarly analyzed to see that faster growing cells are larger than slower growing cells.

As will now be seen, this variation in size is related to, and determined by, the growth rate.

It is proposed that mammalian cells initiate DNA replication at some relatively constant cell size. The time for S and G2 phases are relatively constant as the interdivision time varies [24], so the cell cycle age at initiation of S phase occurs earlier and earlier within the cell cycle as the growth rate increases (or as the interdivision time decreases). This is shown in Fig. 9b, where the interdivision time is varied but S- and G2-phase lengths are constant. In Fig. 9c the cell cycle patterns in Fig. 9b are normalized to a constant length. In Fig. 9c it is clear that the faster cells initiate S phase earlier in the cell cycle. This is because faster growing cells have a relatively short G1 phase. These faster growing cells achieve the initiation mass earlier in the cell cycle and thus these cells will be larger. As in bacteria, *faster growth leads to larger average cell sizes*. (For a discussion of the case of cells growing so fast as to not have a G1-phase as in cell "g" in Fig. 9b, see [24]).

The rate of cell growth is determined by medium composition. For example, as more and more nutrients are added to a minimal medium, bacterial cells grow at faster and faster rates. The interdivision time shortens as the medium becomes richer. For bacteria the mechanism for growth rate variation with medium composition is, in outline, well understood [25]. The addition of nutrients to a medium represses the synthesis of enzymes that are not now needed (e.g., addition of leucine stops the synthesis of leucine synthesizing enzymes). This leads to a shift in the synthetic capacity of the cell to the protein synthesizing system (RNA polymerase, ribosomes, related materials, etc.) as these functions are not repressible by external components [25]. This leads to a more rapid rate of mass increase and thus a shorter mass doubling time [24,26].

Although the details may vary, it is proposed here (and in fact supported by the experiments of Conlon and Raff) that the richer a medium is (e.g., more serum rather than less serum), the faster the cells will grow. The faster a cell grows, the larger it will be (Fig. 9). The variation of G1-phase length with interdivision time variation has been analyzed in detail [24,26].

Conlon and Raff [1] supply evidence for the relationship of cell size and growth rate in their Fig. 7. Cells that have become overcrowded by not being diluted back (their Fig. 7b) decrease their volume (their Fig. 7a).

The analysis presented above explains the variation of cell size as function of growth rate as observed by Conlon and Raff (slower growing cells are smaller than faster growing cells). Furthermore, the analysis can also explain the maintenance of cell size, even with exponential mass increase during the division cycle, as shown in Fig. 9. Larger than average cells will divide sooner as they reach the initiation mass earlier and smaller than average cells will delay initiation until the initiation mass is achieved. Cell division will follow after relatively constant S- and G2/M-phases. This is the underlying and fundamental explanation for the patterns described in Figs. 1 and 2.

Thus, we now have an answer to the question (raised in discussion of Fig. 2) "How does the cell 'know' when to divide so that size homeostasis is maintained?" The answer is that initiation of S phase is determined by the cell mass. A relatively large cell initiates S phase earlier than a relatively small cell. This earlier initiation is played out in an earlier cell division after a period equal to the S and G2/M phases. Since the S and G2/M phases are relatively invariant, an earlier initiation produces an earlier cell division. While the analysis in Fig. 1 discussed the size maintenance problem in terms of the cell dividing earlier if a newborn cell was larger and later if a newborn cell was smaller (or if the rate of mass increase was high or low), the deeper analysis presented now proposes that the decision to divide is determined not at the moment of cell division but earlier at the start of S phase. The initiation of S phase is determined by cell size and the faster a cell reaches the S-phase initiation size the earlier the cell will initiate S phase and the earlier it will divide. For reasons not yet understood, there is a relationship between initiation of S phase and cell division such that once S phase is initiated the cell will ineluctably proceed to division.

We now see the answer to the problem of cell size at division. Cell size at division is merely a surrogate indicator of cell size at initiation. Further, the time of cell division is a surrogate measure of the time of initiation of S phase. A cell that initiates S phase earlier in the cell cycle will have more time to increase its total mass prior to division. The larger newborn cell, having initiated S phase relatively early compared to its relatively smaller sister and cousin cells, will divide earlier as described in Fig. 1. Conversely, smaller cells will delay initiation of S phase; that delay will allow more mass increase before the actual cell division because S phase is somewhat delayed and thus division is postponed allowing mass to increase before the ultimate cell division. In this way, the cell size distribution is maintained.

### Size variation during a shift from slow to fast growth

**Figure 10:**
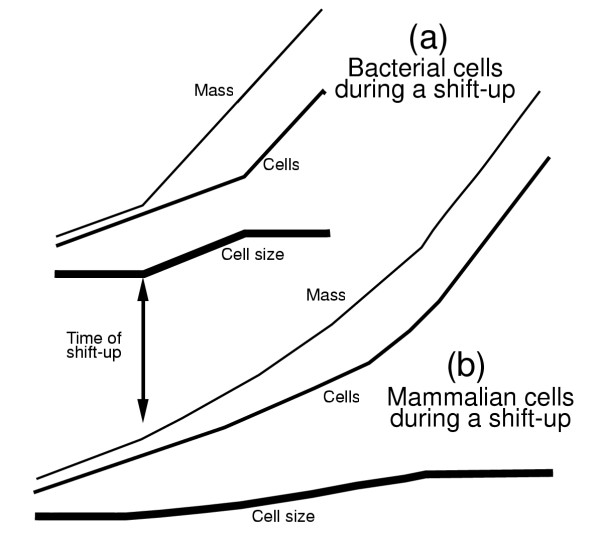
Comparison of shift-up of bacterial cells and mammalian cells. In the panel (a), after a shift of bacterial cells from slow-growth medium to fast growth medium there is an immediate change in the rate of mass synthesis to the new rate while the rate of cell division continues at the old rate for a fixed period of time (rate maintenance). At the end of this "rate maintenance" period, there is a sudden shift in the rate of cell number increase to the new rate. The thick line in panel (a) shows the change in cell size following the shift-up. In contrast, in panel (b) a slower and more gradual change in the rate of mass synthesis, concomitant with the cell number pattern also changing slowly over a period of time, will give a longer period of change in cell size. Conlon and Raff observed this slow pattern of mammalian cell size change.

Immediately following the discovery of bacterial cell size variation with growth rate [8] shift-up experiments of cells from slow growth (relatively small size) to faster growth (relatively large size) were performed [27]. The phenomenon of "rate maintenance" was discovered in this shift-up experiment. Rate maintenance is the continuation of the rate of cell division for a constant period after the shift-up [19]. The rate of mass increase changes immediately to the new rate at the instant of shift-up, while the rate of cell division continues for a period of time before *abruptly *changing to the new rate. The rate maintenance phenomenon occurs over a wide range of shift-ups [19]. The continuation of the original, slower rate of cell number increase, combined with an immediate transition to the new rate of mass increase, leads to an increase in cell size over the period of rate maintenance (Fig. 10a). Rate maintenance is now understood to result from the constant S and G2 periods (C and D periods in bacteria) that do not allow new divisions to occur until the newly inserted replication forks pass through the S (i.e., C) period and the G2 (i.e., D) period. Without going into details here (see [12] for a complete analysis and explanation), suffice it to say that the rate maintenance phenomenon leads to the observed variation in bacterial cell sizes as the rate of cell growth varies over a wide range.

Conlon and Raff [1] studied mammalian cells during a shift-up from slow to rapid growth and small to large cell size. Upon shifting slow cells to faster medium (e.g., shifting cells from low serum to high serum) there is a concomitant increase in cell size (Fig. 6e of Conlon and Raff [1]). One major difference from the bacterial shift-up result is that with animal cells the time for cell size to increase took a much longer time, between 6 and 9 days. To explain the difference between the bacterial shift-up result (Fig. 10a) and the mammalian cell shift-up result (Fig. 10b) one can postulate that for reasons unrelated to the cell cycle but merely related to cellular metabolism occurring continuously throughout the cell cycle, the change in external conditions does not immediately lead to the new rate of mass increase (Fig. 10b). The rate of mass increase is predicted to change relatively slowly as mammalian cells are shifted from serum-free (slow growth) medium to serum-containing (fast growth) medium. Of course, this is just the result reported by Conlon and Raff (their Fig. 6e).

This view of the change in cell size following a shift from slow to rapid growth is quite different from the description Conlon and Raff present for the case of yeast cells switched from a nutrient-poor to a nutrient-rich medium. They write [1], "When switched from a nutrient poor medium to a nutrient-rich medium, the cell cycle arrests and resumes only when the cells have reached the appropriate size for the new condition, which occurs within one cell cycle...Thus, the cells can adjust their size threshold rapidly in response to changing external conditions." The bacterial model of the shift-up allows a rapid change in cell size within one cell cycle without postulating any "arrest" of passage through the cell cycle.

Rather than postulate a mechanism that slows or actively shuts down the cell cycle, it is proposed that no change in cell division occurs until the increased initiations of S phases pass through the S and G2/M phases, as in the bacterial model. No additional mechanism need be proposed to "stop" some event of the cell cycle until cell size has increased.

### The age-size distribution summarizes size control

**Figure 11:**
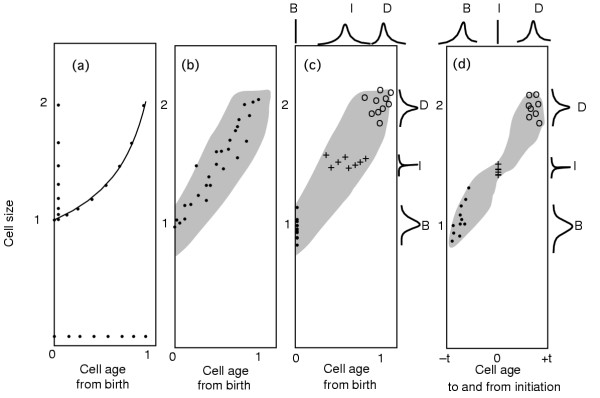
Age-size structure of a growing culture. Panel (a) is the age-size structure for a perfectly deterministic population growing exponentially in mass during the division cycle. The dots on the exponentially increasing line are placed at equal age intervals shown by their representation at the bottom of the panel. The representation of the dots at the left of panel (a) indicates that there is a greater concentration of smaller cells than younger cells. In panel (b) the age-size structure for a population with variation in size and interdivision times is illustrated. The cloud of points (indicated by a few points as representative of the population) is one possible age-size structure. In panel (c) the newborn cells are indicated by the filled circles, the dividing cells by open circles, and the cells in the act of initiation of DNA synthesis by + signs. It can be seen that the larger cells at birth will, on average, reach the size required for initiation of DNA replication more quickly than smaller cells. This is because the larger cells are closer to the initiation size (represented by I on the right side of panel (c)). The B and D distributions at the right of panel (c) indicate the size distributions of newborn (B) cells and dividing (D) cells. The B, D, and I distributions at the top of panel (c) illustrate the age distributions for newborn, dividing, and initiating cells. The size distribution of initiating cells is drawn with a narrower distribution. Variations in mass increase during the period after initiation lead to the widening of the size distribution at division. Panel (d) is a replotting of the pattern in panel (c) with the bottom time scale defined by setting the time of initiation of DNA synthesisas age 0.0. Cells before initiation have a negative age value, and cells after initiation have a positive age value. Initiation takes place, by definition in this panel, at age 0.0. There is some variation in the size of cells at initiation, but it is proposed that this variation is less than the variation at other events of the cell cycle. The narrowing of the age-size structure at the time of initiation is a graphic representation of the size-homeostasis mechanism. No matter what size cells are present at birth or division, these cells are returned to their proper age-size relationship at the instant of initiation of DNA synthesis. Larger cells at division produce larger newborn cells which then reach initiation size earlier than smaller cells which were produced by the division of smaller dividing cells. This is a restatement of the idea that larger cells get to initiation earlier because larger cells have less of a negative age value at cell birth. At the top and right panels of (c) and (d) are representation of the presumed variation of the sizes and ages of cells at particular events. The size at birth is always a little more widely distributed than the size at division due to a slight inequality of partition of mass at division. The size at initiation of DNA replication is drawn with a relatively small variability.

One way to consider a growing culture is to see that every cell in a growing culture has an age and a size. The age/size structure of a population is a representation of each of the cells in the culture and its age and size. If there is no statistical variation, and cells move through the cell cycle with a perfectly precise exponential growth pattern, then the age/size distribution is seen in Fig. 11a. The projections of the dots in this panel to the age axis (bottom, abscissa) and the size axis (left, ordinate) indicate that when cells are growing exponentially, there are a greater number of smaller cells relative to the population than there are younger cells. This reflects the age distribution discussed above.

There are, however, statistical variations in cell sizes at a given age and cells of a given age may have different sizes. The precise statistical distribution is not known, but one view of the possible result is shown in Fig. 11b. The shaded area indicates a cloud of points preferentially collected around the middle of the shaded area, with fewer cells at the outer edges. If this were a three-dimensional graph, there would be a peaked "ridge" up the center of the shaded area indicating that more cells reside with a particular age/size distribution than those at the edges of the age/size distribution.

It is possible to indicate the cells at particular times during the division cycle such as birth, division and initiation of DNA synthesis, as shown in Fig. 11c. There is no distribution in age at birth, since by definition age at birth is 0.0. The graphs at the upper and right sides of Fig. 11c are representations of the spread of the various distributions. There is variability in the age at initiation of DNA replication (I) and the age at division (D). The age at cell birth (B) is defined as constant (i.e., 0.0) but the size at birth (B) is not constant. It is expected that the size at division will be slightly less variable than the size at birth. This is attributable to the probability that any deviation from equipartition of dividing cells leads to a broadening of the size distribution in newborn cells. From Fig. 11c it can also be seen that larger newborn cells (distribution B) will reach the size at initiation (I) earlier than smaller newborn cells. This explains the size maintenance pattern drawn in Fig. 2. Note that in Fig. 11c the size at initiation is relatively narrow. It is this narrowing of size at initiation that leads to the slightly narrower size at division relative to the wider size distribution at birth.

An instructive way of looking at the age/size distribution is to replot cell ages using the age at initiation of DNA synthesis (I) as a starting point (Fig. 11d). By defining age at initiation as 0.0 one gets negative ages for cells before initiation and positive ages for cells after initiation. Fig. 11d shows that there is no distribution in the age at initiation (since by definition the age at initiation is 0.0 for all cells) but there is now a variation in the "age" of newborn cells. Smaller cells have a more negative age and take longer to reach initiation size than larger newborn cells; that is why there is a distribution of cell ages of newborn cells with respect to the time until initiation. The "bottleneck" at initiation of DNA synthesis enables cells born of different sizes to retain size homeostasis. Since all cells to the left of initiation must pass through the bottleneck of initiation on the way to division, all cells, of any newborn size, are realigned and assigned a new age and a new size as they pass through the act of initiation.

## Summary

### Understanding mammalian cell size control

This analysis explains how cell size is maintained through a combination of interdivision time variation and cell growth rate variation. Exponential growth is possible and allowed during the division cycle, in contrast to the proposal of Conlon and Raff [1]. The ideas presented here are a fresh way to look at the cell cycle and cell growth in eukaryotic cells, even though the ideas have been around for over three decades due to work on size determination in bacteria (Cooper, 1979; Helmstetter, 1969). The model of the cell cycle presented here explains many experimental results without postulating checkpoints, G1-phase events, restriction points, or similar phenomena. Experimental support for these ideas [28,29] and the application of these ideas to other problems of cell growth and differentiation [3,13-15,26,28-35] have been published. These ideas have also been reviewed [36-39].

It may be best to summarize these two contrasting views of size maintenance by looking at cell growth in a simple manner, and asking how the rate of mass increase is related to the passage of the cell through the cell cycle. The model of Conlon and Raff looks at the events of passage through the cell cycle as occurring independently of mass increase. The problem then remains as to how mass increase fits into, or coordinates with, the pattern or timing of passage through the cell cycle. It is as though the cell moves through the cell cycle without considering the mass problem, and then the mass of the cell looks at the cell cycle and says "I must grow at some rate so that I do not get too big or too small." In the Conlon/Raff model, a control exists that coordinates mass increase with the rate of cell division.

The model presented here–in contrast to the model of Conlon and Raff–situates mass as the driving force of the cell cycle. Mass increases at some rate that is determined by external conditions (medium, growth factors, pH, etc.). As the mass increases, the accumulation of mass starts or regulates passage through the cell cycle. A cell cannot grow to an abnormal larger size because at a certain cell size or cell mass the S phase is initiated and this event starts a sequence of events leading to mitosis and cytokinesis. A cell cannot get too small because if mass grows slowly (or even stops growing) then the later events of the cell cycle (S-, G2-, and M-phases) are delayed (or do not occur) until mass increases sufficiently to start S phase. A cell cannot get too large because at a certain size the cell initiates S phase leading to the relatively early cell division. A cell cannot get too small because if mass accumulation is inhibited then S phase initiations are also inhibited.

Thus, there is no problem relating mass increase and the cell cycle. Cell mass growth and cell cycle passage cannot be dissociated because one (mass increase) is the determinant of the other (S-phase initiation). For this reason one needs neither checkpoints nor control elements outside of mass increase.

The time for mass to double in a particular situation determines the doubling time of a culture. This is because initiations of S phase occur every mass doubling time, and cell divisions similarly occur every mass doubling time. Thus total mass increases at the same rate as total cell number.

The model presented here explains size determination, size maintenance, and the relationship of mass increase and cell number increase in a growing, exponential, unperturbed, mammalian cell cultures.
